# Primary Rifampicin Mono-Resistant Extrapulmonary Tuberculosis Presenting as a Posterior Chest Wall Abscess: The World’s First Case

**DOI:** 10.7759/cureus.48362

**Published:** 2023-11-06

**Authors:** Sankalp Yadav

**Affiliations:** 1 Medicine, Shri Madan Lal Khurana Chest Clinic, New Delhi, IND

**Keywords:** cbnaat/ xpert/ rif assay, primary extrapulmonary tuberculosis, tuberculosis, rifampicin mono-resistance tuberculosis, mycobacterium tuberculosis (mtb)

## Abstract

Extrapulmonary tuberculosis is a relatively infrequent occurrence. Tubercular involvement of the chest wall is rare. There has never been a record of an isolated primary rifampicin mono-resistant extrapulmonary tuberculosis presenting as a posterior chest wall abscess in the medical literature, making such cases extremely uncommon. In this case report, a 17-year-old Indian male is presented who reported chief complaints of a painless swelling in the posterior chest wall. A comprehensive lab workup ultimately resulted in a conclusive diagnosis. Following the national guidelines, the patient was started on an anti-tubercular treatment.

## Introduction

Tuberculosis poses a significant public health challenge in developing nations [1]. Rifampicin mono-resistant tuberculosis is a type of tuberculosis where the bacteria are resistant to rifampicin. There has been a significant and swift increase in the occurrence of drug-resistant tuberculosis worldwide [2]. While the primary emphasis is typically on pulmonary tuberculosis, sporadic instances have been documented in the literature where the disease has spread through the bloodstream or lymphatic system, or developed locally at extrapulmonary sites [3]. Tuberculosis affecting the chest wall represents a relatively small percentage, accounting for 1-5% of all cases of musculoskeletal tuberculosis. Musculoskeletal tuberculosis itself is less common compared to pulmonary tuberculosis, comprising only 1-2% of all tuberculosis cases [4]. A case of primary rifampicin mono-resistant extrapulmonary tuberculosis presenting as a posterior chest wall abscess is exceedingly rare and never documented in the literature.

A 17-year-old male from India came with the primary concern of a painless swelling on the left posterior chest wall. A diagnosis was confirmed using a cartridge-based nucleic acid amplification test of the pus. In accordance with prevalent guidelines, the patient was initiated on an anti-tubercular treatment plan.

## Case presentation

A 17-year-old non-diabetic Indian male presented to the outpatient department with a painless swelling in the posterior chest wall. Three months ago, the swelling measured 1 x 1 cm, but it grew over time to its current size of 7 x 7 cm. There was no history of fever, cough, weight loss, or loss of appetite. Also, there was no history of trauma or any major medical (including tuberculosis) or surgical intervention in the past. He was a student who had no prior record of substance abuse, detention, or staying at night shelters or refugee camps.

**Figure 1:**
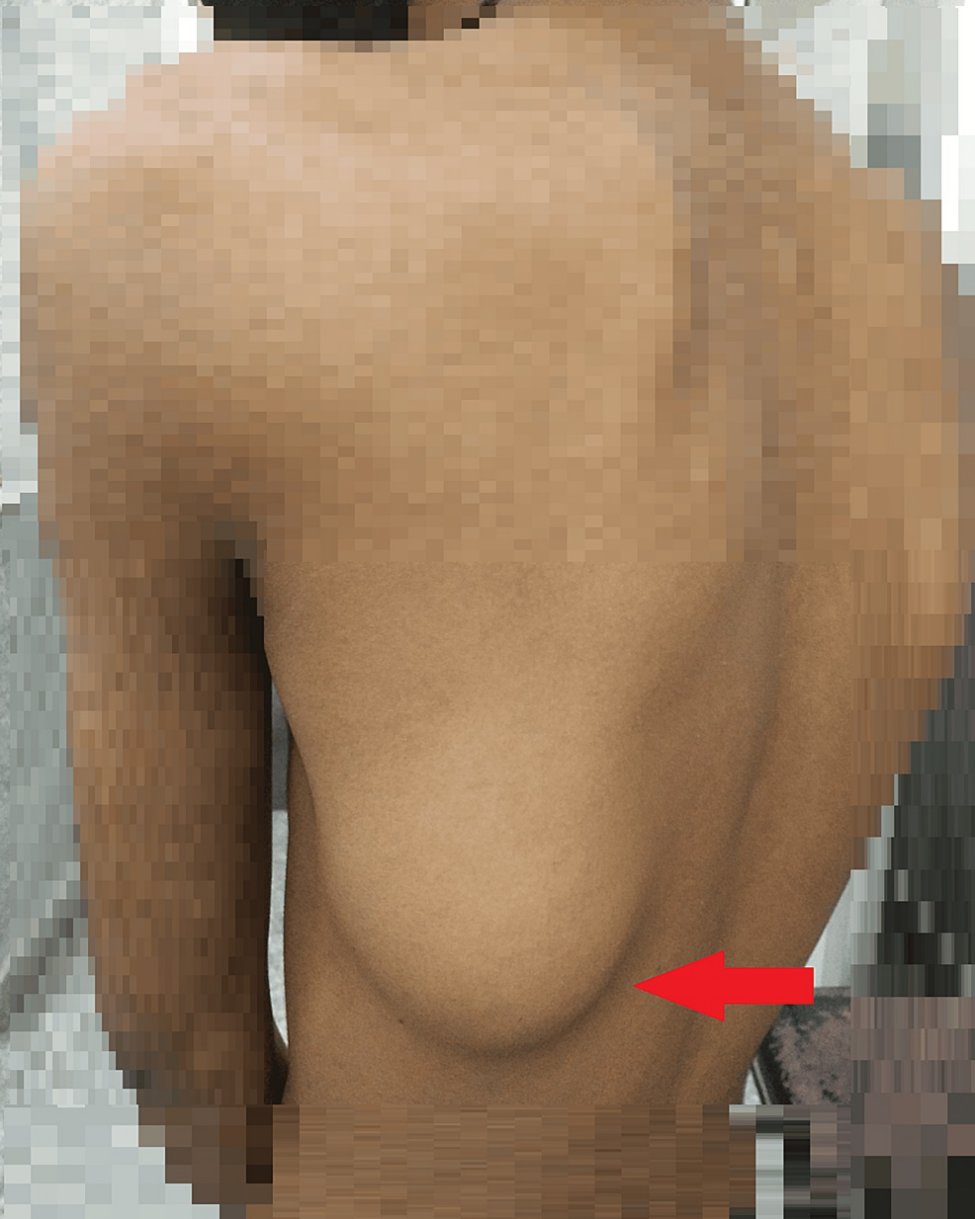
Gross image showing the large swelling

During the examination, the patient was found to be thin-built, afebrile, and hemodynamically stable. An assessment of the respiratory system revealed no abnormalities. However, the local examination unveiled a sizable singular swelling on the posterior chest wall about 3 cm from the infrascapular region measuring 7 × 7 cm. This swelling was soft, non-tender, and warm to touch, with distinct boundaries, mobile, and not adherent to the basal bony structures (Figure 1).

There were no discharging sinuses or engorged veins. Furthermore, there were no signs of icterus, edema, lymphadenopathy, clubbing, cyanosis, or pallor observed during the examination. The assessments of the pulmonary, vascular, abdominal, and neurological systems revealed no noteworthy findings.

Considering the possibility of a pyogenic abscess with differentials, including a tuberculous abscess or tumor, a comprehensive laboratory and radiometric evaluation was scheduled. The patient underwent a standard blood workup and additional examinations, such as sputum smear microscopy and cartridge-based nucleic acid amplification tests of the induced sputum (after inhalation of nebulised sterile saline solution followed by coughing and expectoration of airway secretions).

**Figure 2:**
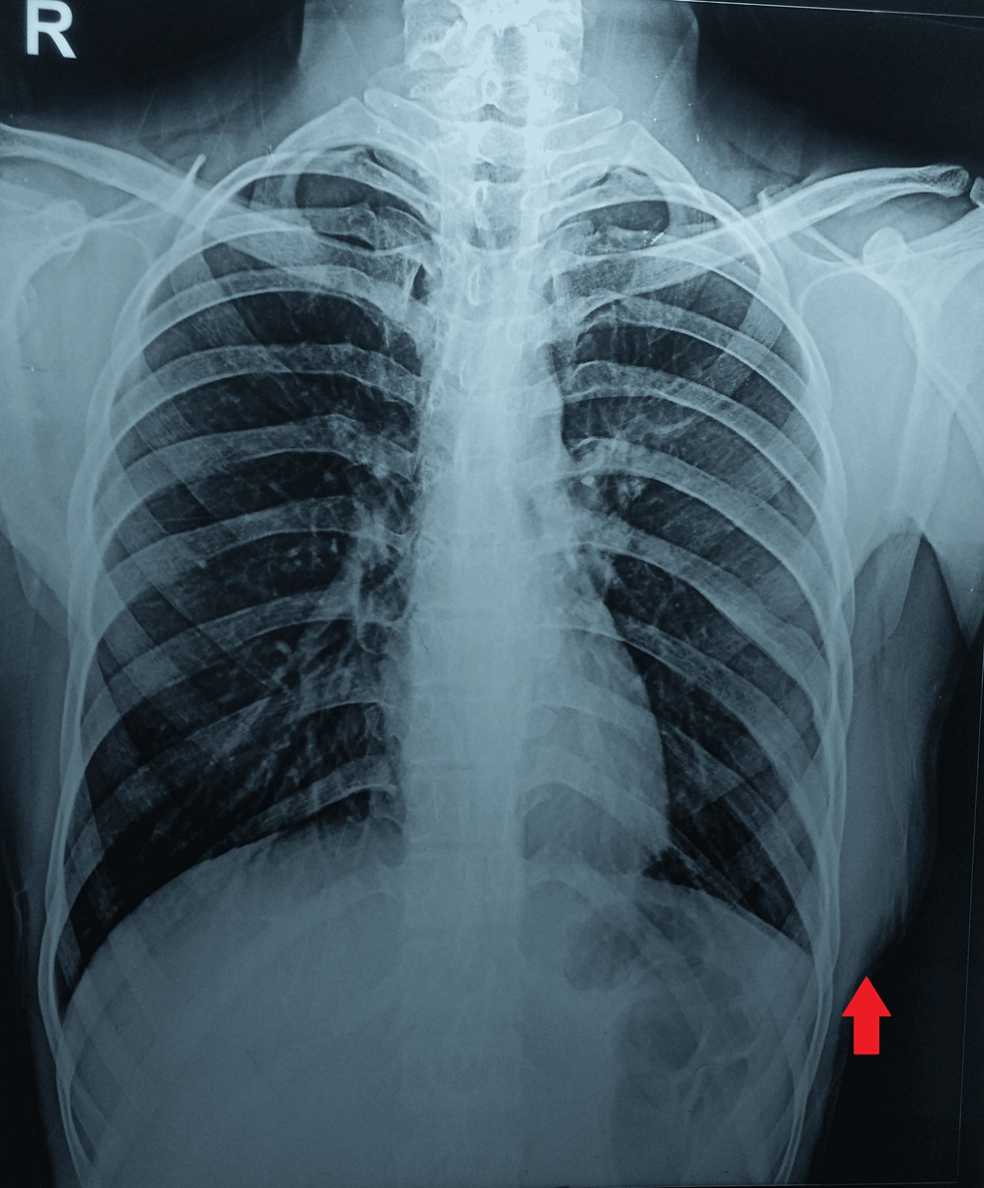
A plain chest radiograph The radiograph showed no pulmonary involvement but indicated the presence of soft tissue swelling.

According to the patient's blood tests, his hemoglobin level was 11.1 g/dL, and his erythrocyte sedimentation rate increased, measuring 63 mm in the first hour. The findings of the rheumatoid factor, hepatitis panel (A, B, and C), and human immunodeficiency virus (HIV) I and II tests were all negative. On the other hand, a very strong positive reaction measuring 30 mm was seen in the Mantoux test. A plain chest radiograph did not reveal any signs of pulmonary involvement but did indicate the presence of soft tissue swelling (Figure 2).

**Figure 3:**
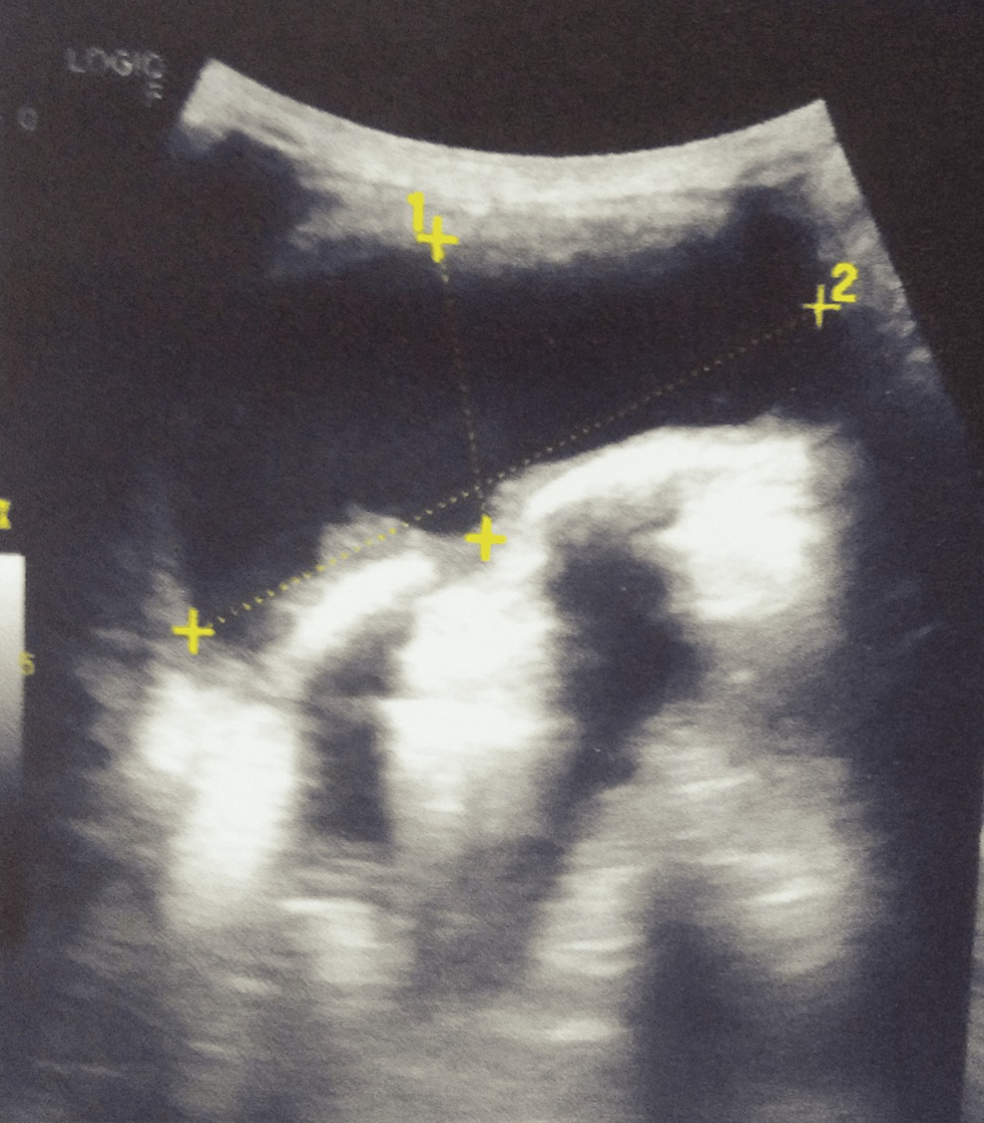
Ultrasonography image of the chest Ultrasonography of the chest showing loculated collection (approximately 52 cc).

An ultrasonography of the chest and axilla was suggestive of a thick-walled anechoic collection with internal echoes in the paraspinal soft tissues in the infra-axillary region on the left side, measuring approximately 28 x 67 x 51 mm (volume of approximately 52 cc) (Figure 3).

Histopathological analysis revealed a significant region of caseous necrosis, encircled by epithelioid cells, with few Langhans giant cells. Chronic inflammatory cells, primarily consisting of plasma cells and lymphocytes, were distributed throughout the sample. Pus tested negative for acid-fast bacilli using the Ziehl-Neelsen staining method. Nonetheless, a cartridge-based nucleic acid amplification test of the pus identified the presence of *Mycobacterium tuberculosis *(low) and indicated resistance to rifampicin. The results from both the line-probe assay and the pus culture returned negative findings.

The ultimate diagnosis was obtained of a primary extrapulmonary posterior chest wall tuberculosis abscess without pulmonary foci that were resistant to rifampicin. The patient was then scheduled for a pre-treatment assessment in preparation for commencing the all-oral, longer conventional regimen as outlined in the national guidelines [5]. Subsequent to an uneventful pre-treatment evaluation, the patient was initiated on treatment, as detailed in Table 1.

**Table 1:** All-oral longer regimen per the patient's weight OD: Once a day; AD: Alternate days.

Drug	Route of administration	Dose	Duration
Bedaquiline	Per oral	400 mg X OD and then 200 mg AD	24 weeks (2+22)
Linezolid	Per oral	600 mg X OD, followed by 300 mg X OD	18 months (6+12)
Moxifloxacin (high dose)	Per oral	600 mg X OD	540 days
Clofazimine	Per oral	100 mg X OD	540 days
Cycloserine	Per oral	500 mg X OD	540 days

The patient displayed excellent tolerance to the anti-tubercular medications, with no notable adverse pharmacological reactions. The limitations of the case were the unavailability of computed tomography of the chest, which was not done due to financial constraints, the non-availability of free spots in government hospitals, and patient refusal. Nevertheless, a conclusive diagnosis was already established, and the patient was initiated on appropriate treatment with regular follow-ups.

## Discussion

Tuberculosis is a significant global public health risk, particularly in developing nations, primarily attributed to extensive immigration, malnutrition, and the prevalence of HIV infection [6]. Chest wall tuberculosis, though infrequent, remains a complex issue in terms of diagnosis and treatment. A defining feature of chest wall tuberculosis is the occurrence of cold abscesses, characterized by swelling devoid of inflammation. This manifestation can manifest as a standalone lesion, unrelated to any primary site in the lung tissue or ribs [7].

The chest wall can be affected by tuberculous abscesses in a number of locations, such as the rib shafts, costovertebral joints, sternum, and vertebrae [7]. These abscesses are most commonly located at the edges of the sternum and along the rib shafts [8]. In approximately half of the cases, multiple lesions can be observed across the chest wall, primarily due to the host tissue's weakened immunological response [7].

Diagnosing this condition presents a significant challenge, especially when dealing with chest wall tumors, other pyogenic wall infections, and actinomycetes infections. It frequently serves as a strong indicator of advanced and widespread tuberculosis. The presentation can be either isolated or linked to pleuropulmonary involvement [9]. Chest wall tuberculosis can develop through two distinct mechanisms, i.e., hematogenous dissemination, often linked to the reactivation of a latent tuberculous focus, direct transcutaneous inoculation, or direct extension, which occurs when tuberculosis spreads from preexisting lymphadenitis in the chest wall [7,9].

In Burke's comprehensive research, the progression of a cold abscess in the chest wall was elucidated through a series of well-designed experimental and anatomical studies [10]. The sequence unfolds as shown in Table 2.

**Table 2:** Progression of a cold abscess in the chest wall Reference [10]

The sequence of development of a cold abscess
*Mycobacterium tuberculosis* infiltrates the pleural space, initiating localized or extensive pleuritis.
From the pleural area, some of these bacilli are transferred to adjacent parasternal or posterior intercostal lymph nodes.
Within these lymph nodes, the tuberculosis bacilli induce the formation of caseous lesions, eventually leading to their rupture.
The necrotic and caseous material from the lymph nodes then migrates anteriorly or posteriorly, forming a cold abscess within the chest wall.

Tuberculosis affecting the chest wall is typically diagnosed through clinical assessment, which involves evaluating general or pleuropulmonary symptoms and examining radiographic findings. Confirmation of the diagnosis often relies on bacteriology and/or histological data [9]. In the present case, a definite diagnosis was achieved with fine needle aspiration cytology and a cartridge-based nucleic acid amplification test of the pus. Cartridge-based nucleic acid amplification testing (GeneXpert®) has emerged as a swift and efficient diagnostic method for tuberculosis, offering significant advantages over microscopy, which tends to have low sensitivity and commonly requires a lengthy culture period [11]. This advanced technique facilitates the early identification of tuberculosis cases, enabling prompt treatment initiation and interrupting the transmission chain. Furthermore, it is capable of detecting resistance to rifampicin [9,11]. Advanced radiometric techniques like computed tomography and magnetic resonance imaging are highly sensitive in determining the extent of infection or any bony involvement. However, in resource-poor settings, they are not always available.

The management of chest wall tuberculosis remains a subject of debate. Some studies have demonstrated positive outcomes when treated solely with anti-tuberculous drugs. On the other hand, opposing results in other series have demonstrated that even with the application of suitable medical care, abscesses were not successfully treated and, in many cases, even deteriorated or resurfaced [9]. Cho et al. have suggested a treatment strategy that involves both preoperative and postoperative tuberculosis medication, as well as the complete removal of the chest wall mass, including any ribs with suspicious involvement [12].

Further delays in making the diagnosis could result in severe complications [10]. Therefore, it is imperative to promptly commence treatment based on drug susceptibility testing for all such cases. Considering the limited available data on this condition, it's essential to recognize that this case represents a single instance. Collecting data from larger medical centers could offer valuable insights for enhancing existing guidelines and facilitating a more targeted approach to managing primary rifampicin mono-resistant chest wall abscesses.

## Conclusions

A case involving a 17-year-old male from India is highlighted in this article. He did not have a history of trauma or tuberculosis; therefore, diagnosing the bulge on the left side of his chest wall was tough. Nevertheless, the diagnosis was eventually confirmed through histopathological examination, a cartridge-based nucleic acid amplification test of the pus, and ultrasonography. Following a pre-treatment evaluation, the patient was promptly started on anti-tubercular chemotherapy. This case underscores the crucial need to prioritize the diagnostic workup and initiation of management in these kinds of unusual clinical situations. Failure to do so can lead to adverse or even fatal outcomes, particularly when healthcare providers lack the necessary knowledge and training.
